# Targeted Strategy in Lipid-Lowering Therapy

**DOI:** 10.3390/biomedicines10051090

**Published:** 2022-05-08

**Authors:** Ezgi Dayar, Olga Pechanova

**Affiliations:** Institute of Normal and Pathological Physiology, Centre of Experimental Medicine, Slovak Academy of Sciences, Sienkiewiczova 1, 813 71 Bratislava, Slovakia; ezgi.dayar@savba.sk

**Keywords:** dyslipidemia, cholesterol, metabolic syndrome, statins, ezetimibe, PCSK9 inhibitors, nanoparticles, targeted therapy

## Abstract

Dyslipidemia is characterized by a diminished lipid profile, including increased level of total cholesterol and low-density lipoprotein cholesterol (LDL-c) and reduced level of high-density lipoprotein cholesterol (HDL-c). Lipid-lowering agents represent an efficient tool for the prevention or reduction of progression of atherosclerosis, coronary heart diseases and metabolic syndrome. Statins, ezetimibe, and recently proprotein convertase subtilisin/kexin type 9 (PCSK9) inhibitors are the most effective and used drugs in clinical lipid-lowering therapy. These drugs are mainly aimed to lower cholesterol levels by different mechanisms of actions. Statins, the agents of the first-line therapy—known as 3-hydroxy-3-methylglutaryl-CoA (HMG-CoA) reductase inhibitors—suppress the liver cholesterol synthesis. Ezetimibe as the second-line therapy can decrease cholesterol by inhibiting cholesterol absorption. Finally, the PCSK9 inhibitors act as an inducer of LDL excretion. In spite of their beneficial lipid-lowering properties, many patients suffer from their serious side effects, route of administration, or unsatisfactory physicochemical characteristics. Clinical demand for dose reduction and the improvement of bioavailability as well as pharmacodynamic and pharmacokinetic profile has resulted in the development of a new targeted therapy that includes nanoparticle carriers, emulsions or vaccination often associated with another more subtle form of administration. Targeted therapy aims to exert a more potent drug profile with lipid-lowering properties either alone or in mutual combination to potentiate their beneficial effects. This review describes the most effective lipid-lowering drugs, their favorable and adverse effects, as well as targeted therapy and alternative treatments to help reduce or prevent atherosclerotic processes and cardiovascular events.

## 1. Introduction

Metabolic disorders are disorders that adversely affect the distribution of macronutrients such as lipids, carbohydrates, and proteins. They are basically a consequence of abnormal chemical reactions in the body that alter the normal metabolic process. While congenital metabolic disorders are caused by genetic defects, acquired metabolic disorders are associated with external factors, such as an unhealthy lifestyle, little physical activity, and excessive caloric intake (for review see [1]). Eckel et al. (2010) documented that human lifestyle is associated with an inherited epigenetic pattern, which affects gene expression, and protein activity that leads to the development of metabolic disorders [2]. Metabolic syndrome is the most common metabolic disorder and represents a cluster of conditions that occur together and increase the risk of heart disease, stroke, and type 2 diabetes. These conditions include increased blood pressure, high blood glucose, obesity, and dyslipidemia [3,4]. Dyslipidemia, manifested by elevated low-density lipoprotein cholesterol (LDL-c), is the primary cause of the development and progression of atherosclerosis. Atherosclerosis is initiated by multiple interactions between oxidatively modified lipids and lipoproteins, inflammatory factors, and components of the immune system in the arterial wall that result in the formation of fatty streaks and fibrous plaques. Plaque buildup and rupture can eventually lead to progressive stenosis and thrombosis. Atherosclerosis may progress silently for a longer period of time until it causes a first cardiovascular event. Analyses of coronary arteries have indicated that dyslipidemia and obesity were predictive of earlier and greater extent of atherosclerosis in large vessels, increase of coronary fatty streaks, and even recurrent coronary events [5,6].

Consequently, dyslipidemia has become a key intervention in the prevention of cardiovascular diseases. In this meaning, therapeutic lifestyle changes and the use of lipid-lowering drugs are the most recommended options for avoiding coronary heart diseases. The main effect of lipid-lowering drugs is the reduction of the plasma low-density lipoprotein or the enhancement of high-density lipoproteins [5,6]. 3- hydroxy-3-methylglutaryl-CoA (HMG-CoA) reductase inhibitors, Niemann-Pick C1-like 1 (NPC1L1) protein inhibitors, and proprotein convertase subtilisin/kexin type 9 (PCSK9) inhibitors are among the most effective and hitherto most used drugs in lipid-lowering therapy. These substances affect respective lipid metabolic pathways and reduce the production or absorption of cholesterol and LDL [7]. However, in addition to their beneficial pleiotropic properties, they have also adverse side effects. Therefore, the goal of a targeted therapy is the reduction of the dose of lipid-lowering drugs while simultaneously achieving a sufficiently effective impact. However, many patients show intolerance to these drugs, so routine treatment needs to be replaced by appropriate alternatives. This review describes the most effective lipid-lowering drugs, their beneficial and adverse side effects, as well as targeted therapy and alternative treatments.

## 2. Routine Lipid-Lowering Therapy

### 2.1. Statins

**Figure 1:**
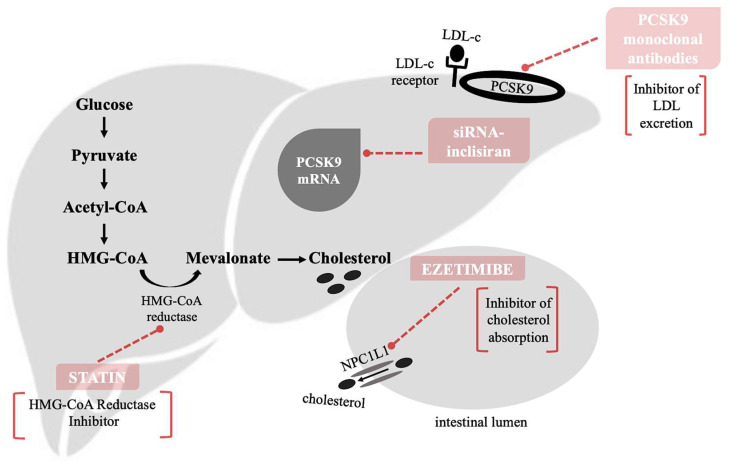
The mechanism by which statins act to reduce liver cholesterol production is based on the competitive inhibition of rate-controlling enzyme in cholesterol synthesis 3-hydroxy-3-methylglutaryl-CoA (HMG-CoA) reductase. This enzyme catalyzes the conversion of HMG-CoA to a mevalonic acid, a necessary step in the biosynthesis of cholesterol. Ezetimibe inhibits the absorption of cholesterol from small intestine leading to the reduction in intestinal cholesterol transmission to the liver [19,22]. The main mechanism is associated with the inhibition of the Niemann-Pick C1-like 1 (NPC1L1) protein, the key factor of cholesterol absorption. Proprotein convertase subtilisin/kexin type 9 (PCSK9) inhibitors inhibit the binding of PCSK9 with low-density lipoprotein (LDL)-receptors and prevent the degradation of LDL-receptors. The inhibition of PCSK9 is targeting also via small interfering RNA (siRNA) against PCSK9 synthesis and expression, small molecules, and vaccination against PCSK9.

Statin therapy represents the gold standard of dyslipidemia treatment. Statins are prescribed as the first-line pharmacological therapy for the reduction of cardiovascular risk [8,9]. The mechanism by which statins act to reduce liver cholesterol production is based on the competitive inhibition of rate-controlling enzyme in cholesterol synthesis HMG-CoA reductase. This enzyme catalyzes the conversion of HMG-CoA to a mevalonic acid, a necessary step in the biosynthesis of cholesterol (Figure 1). Among other products of this pathway are also ubiquinones including coenzyme Q [9,10]. HMG-CoA reductase is active during higher blood glucose level. The basic function of insulin and glucagon is the maintenance of glucose homeostasis. Thus, in controlling blood sugar levels, they indirectly affect the activity of HMG-CoA reductase [11].

Apart from cholesterol-lowering effects, statins also have a wide range of well-documented pleiotropic effects including the improvement of the endothelial function and anti-inflammatory, anti-thrombotic, and immunomodulatory effects for vascular endothelial as well as smooth muscle cells [8,12]. The improvement of the endothelial function is predominantly associated with positive effects of statins on nitric oxide (NO)/reactive oxygen species (ROS) balance, upregulation of endothelial nitric oxide synthase (eNOS) and tetrahydrobiopterin stabilization [8,13,14,15], while anti-inflammatory effects of statins are attributed to their ability to modulate cytokine production [16].

According to the guidelines of the American College of Cardiology (ACC)/American Heart Association (AHA), the dose and the type of statin treatment regimen differ by requiring the reduction of LDL-c rate. However, for all statins, the maximum U.S. Food and Drug Administration (FDA)-approved dose is 80 mg, except for rosuvastatin, which is 40 mg/day. For patients who need a reduction in LDL-c of over 50%, atorvastatin 40–80 mg, simvastatin 80 mg, rosuvastatin 20 mg or combination statin with ezetimibe 10/40 mg are prescribed [17,18]. In spite of highly beneficial effects of statins, they have a low bioavailability. Long-term treatment especially with higher doses may therefore lead to serious side effects such as myopathy, muscle inflammation, joint pains, hemorrhagic stroke, increase of liver enzymes, memory loss, and some neurological disorders [12,19]. The original definition of statin-induced myopathy is the unexplained muscle pain or weakness accompanied by more than 10 times higher creatine kinase concentration. Statin-induced rhabdomyolysis is a severe form of myopathy with more than 40 times higher than the basal activity of creatine kinase and muscle fiber necrosis which often results in myoglobinuria and acute renal failure [20]. In addition, prolongation of statin therapy leads to a decrease in coenzyme Q concentration of up to 40% with corresponding consequences (for review see [21].

### 2.2. Ezetimibe

Ezetimibe inhibits the absorption of cholesterol from small intestine leading to the reduction in intestinal cholesterol transmission to the liver [19,22]. The main mechanism is associated with the inhibition of the NPC1L1 protein, the key factor of cholesterol absorption (Figure 1) located on the gastrointestinal tract epithelial cells and hepatocytes [8,9]. The lower level of cholesterol in the liver cells leads them to absorb more cholesterol from circulation resulting in decrease blood cholesterol level [23].

Ezetimibe was originally developed as a potent inhibitor of acyl-coenzyme A cholesterol acyltransferase to block spontaneous cholesterol efflux, which may be responsible for cholesterol esterification in macrophages [24] and results in formation of intracellular cholesteryl ester, and foam cells [25]. Later it was described as an inhibitor of cholesterol uptake from the small intestine by binding to the NPC1L1 transporter [26].

Major indication of ezetimibe is the elevation of total cholesterol, LDL and apolipoprotein B (ApoB) levels [19]. Ezetimibe also blocks aminopeptidase N and interrupts a caveolin 1–annexin A2 complex involved in cholesterol transport [23]. Latest literature data suggest that ezetimibe may exert several extra-intestinal effects, but these are clearly limited to the inhibition of macrophage migration, reduction of ROS levels and plaque size [8].

Ezetimibe belongs to the class II of antihyperlipidemic drugs and is recommended in second-line therapy for coronary artery diseases prevention after statins [9,22]. Numerous clinical trials showed that ezetimibe could be used either as monotherapy or in combination with statins. It has been used as the first option for patients who are unable to tolerate statins or suffer from their side effects [22,27,28]. The recommended daily dose of ezetimibe 10 mg/day orally is well-tolerated either as monotherapy or in combination with statins or fenofibrates [29].

To date, there is little documented evidence of serious side effects of ezetimibe. Due to insufficient data, ezetimibe is contraindicated and is not recommended for patients with moderate to severe hepatic impairment. However, in monotherapy trials, liver function tests were found to be similar like placebo [28,30]. Studies of Slim and Thompson (2008) and Havranek et al. (2006) have documented the coherence between ezetimibe and myopathy, the second one even pointed to the same effect of ezetimibe and statins in relation to myopathy [31,32].

### 2.3. PCSK9 Inhibitors

PCSK9, a secretory protease, is a member of the proprotein convertase family. PCSK9 is initially secreted as an inactive enzyme precursor which undergoes autocatalytic cleavage in the endoplasmic reticulum of hepatic cells for activation. It moves out of the endoplasmic reticulum to be further handled by the Golgi apparatus before entering the circulation. PCSK9 is secreted into plasma by hepatocytes and has the ability to degrade LDL receptors, inhibiting recycling of receptors to the cell surface (Figure 1). This process thus inhibits uptake of plasma LDL [33,34]. The expression of PCSK9 is induced by sterol regulatory element-binding protein 2 (SREBP-2), leading to LDL-receptor degradation [35]. As result, PCSK9 has become an important cholesterol reduction target. PCSK9 inhibitors inhibit the binding of PCSK9 with LDL-receptors and prevent the degradation of LDL-receptors [36].

There are several strategies that target the inhibition of PCSK9 with different mechanisms such as monoclonal antibodies, synthetic small interfering RNA (siRNA) against PCSK9, vaccination, and small molecules. From human monoclonal antibodies, evolocumab and alirocumab were approved by FDA in 2015 and are currently being marketed. In addition to their ability to decrease LDL-c levels, they may affect the lipid profile by increasing HDL, reducing total cholesterol, and lipoprotein A levels, thus lowering plaque volume. Bococizumab, the third of monoclonal antibodies has been withdrawn by Pfizer while under the evaluation of phase III clinical trials. Although it was able to decrease LDL-c level to 54% [37], after 12-month follow up, it did not demonstrate any benefits regarding the primary end point of major adverse cardiovascular events [38].

Alirocumab is available as a 75 mg/mL pre-filled pen or syringe and is administered every two weeks by subcutaneous injection at a dose of 75–150 mg [39]. Evolocumab is available as a 140 mg/mL single use prefilled syringe or as an autoinjector activated every two weeks. The monthly dose of evolocumab is more than a double of the dose of two-weekly injections because the drug has non-linear pharmacokinetics. Its plasma concentrations do not increase in proportion to the administered dose [39,40]. No adverse effects were reported concerning monoclonal antibodies except injection-site reaction [41] which is related to immunogenicity [42]. It is already accepted that PCSK9 monoclonal antibodies which are already approved have the highest effectiveness comparing long-term statin therapy or statin-ezetimibe combination or ezetimibe alone therapy regimen [27,43,44]. According to the recent studies, monoclonal antibodies have the potential to be used as an alternative to statins [43,45]. However, subcutaneously drug administration, high cost, inaccessibility, and limited long-term clinical outcomes are major obstacles to their wider clinical use [44,46,47].

## 3. Targeted Therapies

### 3.1. Statin-Loaded Nano-Based Drug Delivery System

**Figure 2:**
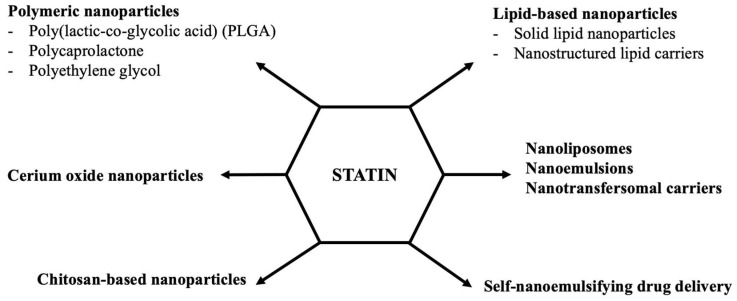
Proposed statin-loaded nanoparticle systems in targeted lipid-lowering therapy.

According to different human studies, long-term statin treatment often causes several adverse effects described shortly in the Part 2 [12,19]. In clinical practice, in such cases, statin doses are reduced, combined with ezetimibe or PCSK9 inhibitors, and/or vitamins or coenzyme Q10 are added to the treatment regimen respectively. In addition, there are several statin-intolerant patients who are unable to tolerate statins at any dose. Considering its low water solubility, rapid metabolism, low bioavailability, and several clinical complications of statins, it is desirable to improve the therapeutic efficacy of the drug and reduce its side effects by developing different therapeutic approaches, such as nanomedicine options [48,49,50]. Today, several nano-formulations, including polymeric nanoparticles, lipid-based nanoparticles, chitosan-based nanoparticles, nanoliposomes, nanoemulsions, nanotransfersomal carriers, self-nanoemulsifying systems, and cerium oxide nanoparticles have been formulated to increase the bioavailability and therapeutic efficacy of statins (Figure 2).

#### 3.1.1. Polymeric Nanoparticles

Among biodegradable polymeric nanoparticles, poly(lactic-co-glycolic acid) (PLGA) which is approved by FDA (for review see [51]) was investigated using different statins. Statin-loaded PLGA nanoparticles displayed a superior profile concerning the bioavailability, drug release, dosing, and minimizing adverse effects [52,53]. In the hyperlipidemic rat model, administration of atorvastatin-loaded PLGA nanoparticles every 3 days exhibited the same efficacy as the once-daily treatment of Lipicure- commercial formulation of atorvastatin calcium. As a result, the daily dose of atorvastatin was reduced by 66% with PLGA formulation [52]. Moreover, pitavastatin-loaded PLGA nanoparticles attenuated the increase of inflammatory inducible nitric oxide synthase (iNOS) activity [54]. Apart from their anti-inflammatory effect, statin-loaded PLGA nanoparticles reduce the progression of hypertension and proliferation of pulmonary smooth muscle cells [55], while exhibiting cardioprotective properties without any adverse effects in different animal models of cardiovascular diseases [56,57]. Similarly, acid-polycaprolactone-based delivery of simvastatin was able to markedly reduce the chemotaxis of vascular smooth muscle cells and intimal hyperplasia [58]. Pitavastatin-loaded PLGA nanoparticles were able to repair injured vasculature via the activation of PI3K signaling pathway promoting the re-reendothelialization and reducing intimal hyperplasia [59]. The increased phosphorylation of Akt [59,60] and eNOS upregulation [54,61] have been mainly responsible for these effects. In agreement with these studies, combined therapy with simvastatin- and coenzyme Q10-loaded polymeric nanoparticles enhanced PI3K-Akt-eNOS pathway by increasing the expressions of Akt and eNOS both in the heart and aorta of obese Zucker rats [62]. Rosuvastatin-loaded poly(L-lactide-co-caprolactone) nanoparticles increased the reendothelialization and reduced the thrombotic potential via increased vascular endothelial growth factor signaling [63]. Later, atorvastatin calcium-loaded poly(ε-caprolactone) nanoparticles were developed and their sustained drug release, improved efficacy, better drug bioavailability, and reduced adverse effects comparing pure atorvastatin have also been confirmed [64,65]. In high-fat diet induced rats, atorvastatin calcium nanoparticles enhanced the NO production and decreased the lipid peroxidation in the liver while simultaneously decreasing the interleukin-1 beta and interleukin-6 levels [66]. Recently, cellulose-based polymer nanoparticles have shown a 3.5-fold enhancement in drug bioavailability. After successful preclinical studies, atorvastatin-loaded ethyl cellulose nanoparticles are considered as a candidate for further clinical trials [49,67].

#### 3.1.2. Chitosan Nanoparticles

Chitosan is known as a biopolymer that coats liposomes to enhance their stability and leads to controlled release of drugs. It increases the nanoformulation efficiency and accessibility of loaded-drug [68]. Chitosan itself may lower cholesterol which has been confirmed in both experimental animal and human studies [69]. Atorvastatin calcium-loaded chitosan nanoparticle formulation showed sustained drug release up to 7 days [70]. In an experimental animal study, rosuvastatin-chitosan nanoparticles were found to be more effective in improving the lipid profile than the pure drug [71]. Recently, novel oral chitosan-based atorvastatin nanocrystals formulation with improved bioavailability have been successfully developed and anti-hyperlipidemic activity has been found to be higher than in marketed Lipitor [72]. In an alternative approach, long-circulating polyethylene glycol (PEG) chitosan nanoparticles showed more than a 72-h drug release profile. Moreover, pharmacodynamic parameters of this formulation were superior to the pure drug [73].

#### 3.1.3. Cerium Oxide Nanoparticles

Cerium oxide nanoparticles have the capacity to eliminate ROS and actually are known as a ROS scavenger. This particular system could accumulate in kidneys and target mitochondria to eliminate excessive ROS [74]. Moreover, cerium oxide nanoparticles interfered with the adipogenic pathway and hindered the accumulation of triglycerides. Transcriptional analysis following in vivo treatment revealed a down-regulation of Lep, Bmp2, Twist1, Angpt2, and Ddit3, and an up-regulation of Irs1 and Klf4 expression. Overall, cerium oxide nanoparticles contributed to a slowing of weight gain and lowered the plasma levels of insulin, leptin, glucose, and triglycerides [75]. In this sense, ceria nanoparticles coated with ROS-responsive organic polymer (mPEG-TK-PLGA) and loaded with atorvastatin demonstrated a greater antioxidant and anti-apoptotic activity than the pure drug, and effectively decreased oxidative stress and inflammatory processes in acute kidney injury mice model [74].

#### 3.1.4. Lipid-Base Nanoparticles

Among the lipid-based nanoparticle formulations, solid lipid nanoparticles (SLN) and nanostructured lipid carrier (NLC) are well-studied promising delivery systems [76]. Comparing free statins, oral bioavailability and absorption of statin-loaded SLNs were markedly enhanced [77,78,79]. A sustained release of simvastatin from the lipid core of nanoparticles was confirmed as well [80]. In high-fat diet induced hyperlipidemic rats, the treatment of atorvastatin-loaded SLNs combined with coenzyme Q10 and vitamin E showed superior effect on lipid profile. Atorvastatin-loaded SLNs alone administration illustrated weaker effect in reducing triglycerides and LDL levels than the combined therapy with coenzyme Q10 and vitamin E [81].

Since the NLCs formulation has produced and evolved from SLNs, several studies compared the effects of drug profile between SLNs and NLCs. According to the results, statin-loaded NLCs exhibited superior pharmaco-technical properties in regards to sustained and gradual drug release than SLNs [76,82]. Moreover, displaying greater results in lowering total cholesterol, LDL, triglycerides, and elevating HDL was observed in comparison with the standard drug [82]. 5-weeks-of-simvastatin-loaded NLCs treatment exhibited the recovery of blood lipid levels, inhibition of smooth muscle cell apoptosis, and the delay in the onset of atherosclerosis in hyperlipidemic rats [83]. Statin-loaded NLCs formulations also exerted prolonged reduction in the total cholesterol and non-high-density lipoprotein cholesterol levels [84]. The pharmacodynamic and pharmacokinetic profile of NLCs with statins has been tested in several studies confirming that this treatment approach could be used to improve the statin oral delivery and bioavailability, clinical efficacy, and anti- hyperlipidemic activity [84,85,86,87].

Statin-loaded HDL nanoparticles showed the inhibition of inflammation progression and a decrease of atherosclerotic plaques while simultaneously achieving these effects without any hepatotoxicity. Injectable reconstituted HDL nanoparticle carrier vehicle was able to deliver statin directly to the atherosclerotic plaques [88,89].

#### 3.1.5. Nanoliposomes, Nanoemulsions, Nanotransfersomal Carriers

In high-fat diet rats, simvastatin-loaded lipid core nanocapsules exhibited greater efficiency than same lovastatin-loaded nanocapsules [90]. In studies with novel nano-formulations indicated that simvastatin-loaded nanoliposomes exerted a higher plasma simvastatin concentration than the pure drug [91] and nanoemulsion of simvastatin promised higher oral bioavailability with almost 100% of drug release [92].

Chen et al. suggested that the lipophilic emulsifier is crucial for the oral absorption of the drug. In their study, the lipophilic emulsifier named Myverol and soybean phosphatidylcholine were used and successfully formulated lovastatin-loaded NLCs. According to the results, lovastatin administration of Myverol-containing NLCs were found to be more stable in the gastric environment compared to soybean phosphatidylcholine. When compared with the free drug, lovastatin-loaded NLCs from Myverol exhibited greater plasma concentrations [85].

In another unique study, the effect of transdermal delivery system was investigated in hyperlipidemic rats. The developed nanotransfersomal carrier exerted potent effects on hyperlipidemia without serious side effects on liver in comparison to an oral atorvastatin treatment. Interestingly, in hyperlipidemic rats, pure atorvastatin had no effect on lipid profile. On the other hand, nanotransfersomal atorvastatin decreased significantly total cholesterol, triglycerides, and LDL-c levels. This pioneer study showed for the first-time amelioration of dyslipidemia by the treatment with transdermal atorvastatin nanotransfersomal gel system. Transfersomes may open a window of opportunity for the well-controlled transdermal delivery of drugs that produce side effects following oral administration [93].

#### 3.1.6. Self-Nanoemulsifying Drug Delivery System

Self-nanoemulsifying drug delivery system (SNEDDS) of statins could be a novel formulation to enhance drug profile. SNEDDS of statins improved the drug dissolution rate [94], increased the oral bioavailability approximately 2.4-fold [94,95] and the expansion of drug release 4-fold [96] compared to the pure statins. Statin-loaded SNEDDS system has exerted remarkable antihyperlipidemic properties by normalizing serum lipid levels [97] and overall, it has a valuable potential to improve the oral absorption as well as the pharmacodynamic efficacy compared to the pure drug [98,99].

According to the previous findings, it is obvious that statin nano-therapy using different nanotechnology systems may contribute to the reduction or elimination of common adverse effects related to the statin treatment and helps statin delivery as well as their positive pleiotropic effects.

### 3.2. Ezetimibe-Loaded Nano-Based Drug Delivery Systems

**Figure 3:**
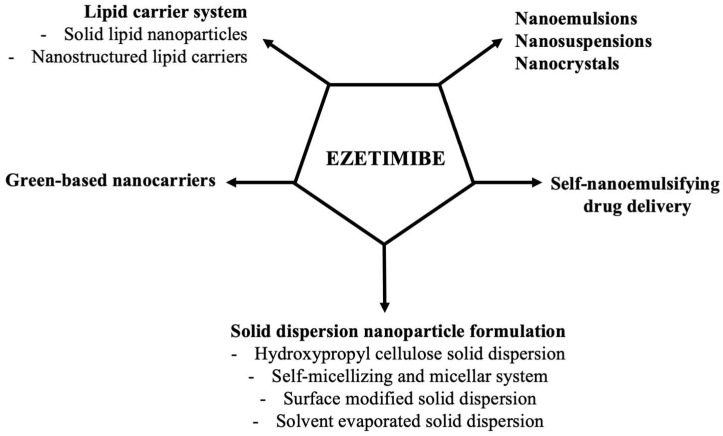
Proposed ezetimibe-loaded nanoparticle systems in targeted lipid-lowering therapy.

According to the Biopharmaceutics Classification System, ezetimibe belongs to the class II/IV of compounds which display a low aqueous solubility and absorption and high permeability resulting in poor bioavailability [100]. In the literature, many approaches can be found concerning upgrade ezetimibe drug profile (Figure 3).

#### 3.2.1. Lipid Carrier Systems

Lipid carrier systems have been documented as suitable delivery systems to increase the bioavailability of ezetimibe. Comparing to marketed product and drug suspension, ezetimibe-loaded SLNs showed greater stability. According to the results, stability of ezetimibe-loaded SLNs remained unchanged for 3 months. However, slow and limited ezetimibe release lead to decreasing its bioavailability [101]. On the contrary, in high-fat diet rats, ezetimibe-loaded NLCs increased drug bioavailability even with reduced dose compared to the pure drug. Simultaneously, triglyceride, HDL-c, LDL-c and cholesterol levels were normalized and liver functions were improved. Ezetimibe-loaded SLNs are ready today for clinical evaluation [102].

#### 3.2.2. Green-Based Nanocarriers

Nowadays, the attention has been drawn to biosustainable and biodegradable systems like green-based nanocarriers. It has been documented that biodegradable nanoparticle adjusted drug therapeutic value by increasing its bioavailability, solubility, and release time [103]. Using linseed mucilage alternative in the development of polymeric nanoparticle formulation, pharmaceutical properties and efficacy of ezetimibe were enhanced. This study concludes that linseed mucilage seems to be a resourceful alternate for the delivery of therapeutic agents with poor water solubility [104].

#### 3.2.3. Nanoemulsions, Nanosuspensions, Nanocrystals

Also, nanoemulsions and nanosuspensions of ezetimibe illustrated better drug absorption than the marketed formulation and belong to possible alternatives in improving the drug profile [105,106]. Ezetimibe nanosuspensions completely dissolved in the dissolution medium within 1 h, while pure drug was dissolved only up to 42% during the same time. Better drug dissolution resulted in improved oral bioavailability of ezetimibe [107]. The drug release profile of nano-ezetimibe from volatile microemulsion template was increased significantly >98% in 30 min [108].

Similarly, nanocrystals of ezetimibe remarkably increased its solubility and displayed a superior pharmacodynamic activity upon oral administration. Solubility, dissolution, and pharmacodynamics in lipid lowering activity using ezetimibe nanocrystals were preferable [109]. Dual drug nanocrystals loaded nano-embedded microparticles with a combination of simvastatin and ezetimibe were also studied. Nanocrystals of both drugs demonstrated a significant enhancement of dissolution in comparison to their physical mixture [110].

#### 3.2.4. Self-Nanoemulsifying Drug Delivery

It has been proven that self-nanoemulsifying granule system of ezetimibe ensured 128% protection while pure ezetimibe can offer only 58%. In high-fat diet rats, this formulation yielded a higher reduction in the total cholesterol levels due to enhancement of drug solubility and absorption. On the other hand, there was no significant difference in the HDL levels after 14-days of treatment, by reason it had no direct action on the blood HDL levels like statins [111]. Great potential of optimized super saturable SNEDDS (S-SNEDDS) has been reported for the first time by Gao and Morozowich (2006) [112]. Later, Bandyopadhyay and Singh (2012) investigated the impact of the same formulation and presented the enhancement of the drug absorption [113].

#### 3.2.5. Solid Dispersion Nanoparticle Formulation

Solid dispersion nanoparticle formulation is another approach to find out an effective oral drug delivery system. Ezetimibe-hydroxypropyl cellulose (HPC) solid dispersion nanoparticle system enhanced absorption and low dissolution rate of pure ezetimibe with about 7.5% increasement in maximum dissolution and 9.9% increasement of dissolution efficiency [114]. Torrado-Salmeron et al. described ezetimibe self-micellizing solid dispersion with a non-ionic emulsifier and solubilizer called Kolliphor. Results showed that serum levels of total cholesterol, LDL, and triglycerides were significantly improved when compared to pure ezetimibe in high-fat induced hyperlipidemic rats [115]. The same group of authors also investigated the comparison between solid dispersion of ezetimibe and micellar system of ezetimibe with Kolliphor. The second one showed greater solubility and better oral bioavailability accompanied by an improvement of the lipid profile with half dose of ezetimibe and using different formulation of micellar system [116].

A comprehensive study that examined the differences between ezetimibe-loaded different formulation approaches such as SNEDDS, surface modified solid dispersion (SMSD), and solvent evaporated solid dispersion (SESD) revealed higher total ezetimibe plasma concentrations and aqueous solubility as compared with the pure ezetimibe. All formulations significantly ameliorated dissolution of the drug in the following order: SNEDDS ≥ SESD > SMSD (200-fold for SNEDDS, 110-fold for SESD, and 80-fold for SMSD). Because SESD gave higher area under curve from zero to infinity than did SNEDDS and SMSD, this system was mostly recommended as a drug delivery system for the oral administration of ezetimibe [117].

Statin combination therapy with ezetimibe may significantly reduce the incidence of severe atherosclerotic events [118]. Indeed, combination of ezetimibe and atorvastatin loaded nano-solid dispersions improved efficiency, serum lipid levels, and reduced the toxic effects of cholesterol on the liver tissue [119,120]. Interestingly, atorvastatin even enhanced solubility of ezetimibe and its dissolution profile. On the other hand, atorvastatin release was decreased in the presence of ezetimibe [120]. Micellar system of ezetimibe and atorvastatin combination therapy using nano solid dispersions with Kolliphor showed significantly greater impact on lipid profile than the same dose of pure drugs [121].

### 3.3. PCSK9 Inhibition Targeting

**Figure 4:**
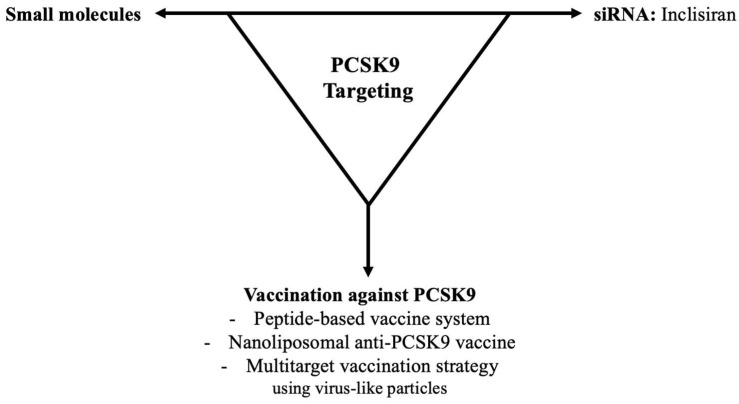
Proposed PCSK9 inhibition targeting in lipid-lowering therapy.

With the exception of monoclonal antibodies, the inhibition of PCSK9 is targeting via siRNA against PCSK9 synthesis and expression, small molecules, and vaccination against PCSK9 (Figure 4). While siRNA is in the phase III clinical stage, the last two brand-new approaches are still in the preclinical study stage [122,123,124].

#### 3.3.1. siRNA: Inclisiran

Inclisiran is the first siRNA-based drug that acts as an inhibitor of PCSK9 expression. It specifically targets and binds to PCSK9 mRNA leading to PCSK9 degradation and resulting in diminished PCSK9 protein levels and long-lasting reduction of LDL-c even up to 3–6 months [125,126,127]. It has a long biological half-life that induced sustained LDL-c lowering, which seems to be more preferable than monoclonal antibody therapy [128].

Inclisiran efficacy, toleration, and its ability to reduce LDL-c levels have been displayed by preclinical studies, Phase I and Phase II clinical trials. Phase III clinical trials are still in progress [126,129]. According to the ORION-1 randomized clinical trial, one or two injections of inclisiran can remarkably reduce PCSK9 and LDL, decrease atherogenic lipids, improve lipoprotein profiles and appear to be safe and well tolerated [130]. Patients with a high cardiovascular risk received one or two doses of inclisiran for 6 months. Their results showed that PCSK9 and LDL-c levels were markedly reduced from the baseline [131]. After one-year-follow-up, patients who received two-dose-inclisiran have still remained the reductions on the PCSK9 and LDL-c levels. According to the results, twice a year administration of inclisiran could maintain to stable reductions in LDL-c levels [132].

Novartis recently introduced inclisiran and received its first approval from the European Union in 2020 for patients with primary hypercholesterolemia and dyslipidemia [128,129]. It could be used with either a statin or a statin-ezetimibe [129]. Among its side effects, injection site reactions, injection site pain, erythema and rash have been reported. Other than these possible adverse effects, such as an increased risk of cardiovascular events or organ toxicity, are still unknown, but are being thoroughly tested in clinical trials [127,129].

In a preclinical study testing the use of lipid-containing nanoparticles with the precursor ALN-PCS it has been shown that this system was able to reduce PCSK9 mRNA and protein concentrations by 70% and LDL-c concentrations by 60%. These effects lasted for 3 weeks after a single intravenous administration in the animal models. This treatment was also able to reduce total cholesterol and apoB levels [133]. A phase I clinical study that observed the effects of ALN-PCS did not show any serious adverse effects. On the contrary, PCSK9 and LDL-c levels were significantly reduced for at least 6 months [134]. According to the meta-analysis of randomized clinical trials, in patients with familial hypercholesterolemia and/or atherosclerosis, the drug found to be effective, safe, and well tolerated at lower LDL-c levels and exerted only mild adverse effects along with injection site reaction [135].

#### 3.3.2. Vaccination against PCSK9

Vaccination strategies could be a better alternative than monoclonal antibodies because their approach is less expensive and does not require frequent administration intervals [136,137]. The peptide-based PCSK9 vaccine has been shown to induce the production of antibodies that improve the lipid profile for up to 24 to 40 weeks, according to data from animal studies [138,139]. According to the study of Landlinger, the PCSK9 vaccine reduced plasma lipids and systemic and vascular inflammation by reducing plasma inflammatory markers and vascular endothelial growth factor leading to weakening of atherosclerotic lesions in the aorta [140]. Similarly, in a peptide-vaccinated mice, an effective immune response was associated with a significantly improved lipid profile [141]. In vaccinated hypercholesterolemic mice, Wu et al. have shown a reduction in total cholesterol and LDL-c [142]. Recently, phase I clinical trial is conducted to assess the safety and tolerability of anti-PCSK9 vaccine strategy in healthy subjects. The results have not been published yet [143].

Momtazi-Borojeni et al. introduced a nanoliposomal anti-PCSK9 vaccine [144] and in the first approach administered it to healthy animals to observe antibody production [145]. After detection of antibody production, immunized rats were administered intraperitoneally with streptozotocin to induce diabetes mellitus. It is reported that vaccinated rats showed lower plasma LDL-c levels compared to non-vaccinated diabetic rats. Hyperglycemia was suppressed in vaccinated rats [146]. In hypercholesterolemic mice, the nanoliposomal vaccine against PCSK9 promoted antibodies that inhibited the interaction between PCSK 9 and the LDL receptor and also led to a reduction in LDL and triglycerides. In addition, a reduction in inflammatory cells as well as a reduction in the size of atherosclerotic lesions have been observed [147]. Ortega-Rivera et al. established technology for a single-dose multi-target vaccination strategy targeting ‘cholesterol checkpoint’ proteins including PCSK9, ApoB, and cholesteryl ester transfer protein (CETP). The candidate vaccine was developed using virus-like particles from bacteriophage Q-beta that exhibit PCSK9, ApoB and CETP antigens. Decreased plasma PCSK9 and ApoB levels, in vitro CETP inhibition and decreased total plasma cholesterol were observed after vaccination [148].

#### 3.3.3. Small Molecule PCSK9 Targeting

Agents of small molecule PCSK9 targeting could be a new alternative of lipid-lowering drugs. They are potentially safer and have more advantages such as small size, low cost, and easier production than the other respective treatments [149,150]. Despite that, development of small molecule PCSK9 targeting is still in the preclinical stage [151].

Shifa Biomedical has developed the first small molecule that targets PCSK9, called P-4. It successfully inhibits PCSK9 and LDL binding and reduces circulating LDL-c levels. Due to the low solubility and permeability in the water, a new nano-formulation P-4 called P-21 has been developed to overcome these obstacles. The P-4 formulation had better plasma bioavailability, than P-21. However, targeted delivery of the P-21 nano-formulation to the hepatocytes showed higher bioavailability in the liver. The LDL-c lowering efficacy of P-21 was about 90% and much higher than that of P-4. In addition, it led to a 2-fold increase in HDL-c after two weeks of oral treatment. The development of a small molecule nano-formulation represents a new alternative to lipid profile modification with the potential for significant benefits for clinical practice [152].

## 4. Conclusions

Nowadays, statins are the key medication for treatment of dyslipidemia in patients who have or are at substantial risk for atherosclerotic cardiovascular diseases. However, with the intensity of statin treatment, intolerance to their use has the increasing tendency. Therefore, new lipid-lowering drugs that interfere with various mechanisms of lipid pathways are being evaluated. Clinical trials have documented that ezetimibe and PCSK9 inhibitors can successfully lower LDL-c level and contribute to lowering cardiovascular events. However, recent studies have shown that also these drugs may have some side effects, or the route of their administration is unsatisfactory. To improve the therapeutic efficacy of the lipid-lowering drugs and reduce their side effects different nanoparticle systems with targeted strategy have been developed and described. Thus, enhancing the pleiotropic effects of lipid-lowering drugs by suitable targeted strategy may represent a promising tool for the treatment of atherosclerotic cardiovascular diseases.
